# An inverse relationship between perceived social support and substance use frequency in socially stigmatized populations

**DOI:** 10.1016/j.abrep.2019.100188

**Published:** 2019-05-20

**Authors:** Rachel Rapier, Scott McKernan, Christopher S. Stauffer

**Affiliations:** a San Francisco VA Medical Center, 4150 Clement St. (116C-1), San Francisco, CA 94121, USA; b Department of Psychiatry, University of California, San Francisco, 401 Parnassus Ave, San Francisco, CA 94143, USA; c The New School, 72 5th Ave, New York City, NY 10011, USA

**Keywords:** Social support, Addictive behavior, Substance-related disorders, Sexual and gender minorities, Prisoners, Social psychology

## Abstract

**Introduction:**

Social isolation and alcohol and substance use disorders (ASUD) have been identified as global health risks. Social support is protective against developing ASUD and is associated with beneficial addiction treatment outcomes. Socially stigmatized populations are at higher risk of both social isolation and ASUD, and the link between social support and substance use in these populations has been less researched than in general substance-using populations. We hypothesized that perceived social support, as measured by the Social Provisions Scale (SPS), would have an inverse relationship with frequency of substance use, from subsections of the Addiction Severity Index (ASI) that estimate use over the past 30 days and over an individual's lifetime.

**Methods:**

Using a cross-sectional design, we conducted secondary correlational analyses with pre-existing data to test our hypothesis in two separate samples made up of socially marginalized populations entering ASUD treatment programs. Sample 1: substance-using male prison inmates (*n* = 72, average age = 30.79) and Sample 2: primary methamphetamine-using men who have sex with men (*n* = 86, average age = 43.41).

**Results:**

Significant negative correlations were found between SPS and lifetime use of alcohol, tobacco, and cannabis (*r*_*s*_ − 0.27, −0.39, −0.26; *p*-values 0.04, 0.001, 0.04, respectively) in Sample 1 and 30-day use of methamphetamine (*r*_*s*_ − 0.28; *p*-value 0.008) in Sample 2.

**Discussion:**

Differences in results between the samples (lifetime vs 30-day use) may reflect psychosocial and contextual differences impacting perceived social support. Our findings provide support for an important link between perceived social support and frequency of substance use in socially stigmatized populations.

## Highlights

• Socially stigmatized populations experience higher prevalence of social isolation and substance use disorders.
• Lower social support is linked with higher lifetime alcohol, cannabis, and tobacco use in a prison inmate sample.
• In a sample of meth using men who have sex with men, lower social support is linked with more meth use over the past 30 days.

## 1 Introduction

Loneliness and social isolation are public health concerns with mortality risks comparable to those of alcohol and substance use disorders (ASUD; Holt-Lunstad, Smith, Baker, Harris, & Stephenson, 2015), and large-scale deficits in social connection are thought to play a role in the rising prevalence of ASUD (Alexander, 2012). Social support is reliably related to lower overall morbidity and mortality (Uchino, 2006) and is protective against the development of ASUD (Stone, Becker, Huber, & Catalano, 2012; Wills, Vaccaro, & McNamara, 1992). Moreover, higher levels of social support have been linked to multiple encouraging addiction-related outcomes, including fewer pretreatment days using alcohol and drugs (Zywiak et al., 2009) and lower frequency of relapse (Atadokht, Hajloo, Karimi, & Narimani, 2015).

Social identity, or aspects of our sense of self that we derive from our social group membership (Jetten, Haslam, Haslam, Dingle, & Jones, 2014), also impacts addiction risk, severity, and recovery. For example, individuals with multiple sources of valued social identity prior to developing ASUD experience addiction as an “identity loss”, whereas individuals who were socially isolated prior to developing ASUD experience addiction as an “identity gain” (Dingle, Cruwys, & Frings, 2015). The Social Identity Model of Recovery (SIMOR; Best et al., 2016) and the Social Identity Model of Cessation Maintenance (SIMCM; Frings & Albery, 2015), characterize the shift from addiction to recovery as an overall increase in social connectedness and a shift in the composition of social networks from substance-using peers to non-using peers, which reflects an important transition in social identity (Bathish, Savic, Beckwith, Mackenzie, & Lubman, 2017; Best et al., 2016).

Marginalized social identities, such as individuals with a history of incarceration or LGBTQ individuals, are particularly vulnerable to experiencing insufficient social support (Biggam & Power, 1997; Rokach & Cripps, 1999; Meyer, 2003; Kecojevic, Basch, Kernan, Montalvo, & Lankenau, 2019) and suffer from ASUD at rates many orders of magnitude higher than the general population (Cochran, Ackerman, Mays, & Ross, 2004; Fazel, Bains, & Doll, 2006). Furthermore, the elevated prevalence of ASUD in prison inmates and LGBTQ individuals has been linked to the experience of social stigmatization (Meyer, 2003; Newcomb, Heinz, & Mustanski, 2012; Moore & Tangney, 2017). While the association between social support and substance use outcomes has been well-established in general, research conducted specifically with marginalized populations is less defined. Thus, the present study aims to investigate the relationship between social support and substance use in socially stigmatized populations.

We reviewed measures of social support and substance use and determined that the following two measures would most optimally align with our aims. The Social Provisions Scale (SPS) (Weis, 1974) is a well-validated and frequently used measure of perceived social support based on Weiss's theory of social provisions. Importantly, perceived social support—referring to one's anticipated access to potential social support—has consistently demonstrated a greater beneficial impact on health than reports of actual received social support (Uchino, 2009), and measures of social support grounded in theory tend to perform better than general measures of support (Gottlieb & Bergen, 2010). The Addiction Severity Index (ASI) is a structured interview that has been widely used in research for almost four decades to assess multiple dimensions of addiction (McLellan, Luborsky, Woody, & O'Brien, 1980; McLellan, Cacciola, Alterman, Rikoon, & Carise, 2006), including standardized methods to quantify frequency of use over the past 30 days and over an individual's lifetime. Lastly, we 1) identified a pre-existing data-set that includes both the SPS and ASI in a sample of prison inmates and 2) collected SPS and ASI data from a separate sample of men who have sex with men (MSM). We hypothesize that perceived social support, as measured by the SPS, of these socially stigmatized populations will be inversely related to 30-day and lifetime frequency of substance use prior to entering addiction treatment.

## 2 Methods

All research methodology for this exploratory analysis was approved by the Institutional Review Board at the University of California, San Francisco.

### 2.1 Participants

We conducted an analysis of data from two separate clinical samples entering treatment for ASUD.

#### 2.1.1 Sample 1

After selecting our target instruments, we identified an existing archival data-set that included both SPS and ASI data (Cadoret, 2003). We obtained the data-set from the National Institute of Justice Data Resource Program for secondary analysis. The data were collected between January 1998 and March 1999 from 441 male inmates during intake to a voluntary, free-of-charge, six-month, residential, addiction treatment program. The study's purpose was to assess the effectiveness of this program at the Clarinda Correctional Facility in Clarinda, Iowa. Assessments were administered by extensively trained counselors. Inmates enrolled in the program were within 12 months of release and were identified as having a need for residential-level addiction treatment. They had been incarcerated for various periods of time prior to beginning the program; thus, ASI-30 examined “typical 30-day use” prior to incarceration rather than actual past 30-day use.

#### 2.1.2 Sample 2

In order to confirm our hypothesis using a separate non-incarcerated sample, we used SPS and ASI data collected from 86 MSM between March 2017 and May 2018 by trained clinicians. All participants identified methamphetamine as their substance of choice and had used at least once in the past 30 days. All participants were recently engaged in voluntary treatment for methamphetamine use disorder at a free community mental health program in San Francisco, California and were being assessed for a separate clinical trial (Stauffer et al., 2019) for which the present study is an ancillary analysis.

### 2.2 Measures

Both the SPS and ASI have good indicators of validity and reliability and have been extensively used by independent research groups (Weis, 1974; McLellan et al., 2006, McLellan et al., 1980; Bastos, Duquia, González-Chica, Mesa, & Bonamigo, 2014).

#### 2.2.1 Perceived social support

The SPS is a 24-item self-report measure of current perceptions of social support using a 4-point Likert scale. We used the SPS global score (range 24–96), which has excellent internal consistency reliability (α = 0.93) (Cutrona & Russell, 1987). No previously published studies using the SPS have reported on the relationship between perceived social support and substance use frequency.

#### 2.2.2 Frequency of substance use

The ASI, a semi-structured clinical interview, includes a portion that assesses alcohol and drug use frequency (McLellan et al., 1980). To test our hypothesis, we used the following calculations: i) (number of days of use in the past thirty days)/30 (ASI-30) and ii) (number of years using three or more days per week over the individual's lifetime)/(individual's age in years) (ASI-LT), both presented as ratios between 0 and 1. The entire ASI was administered to Sample 1, but only ASI-30 and ASI-LT were administered to Sample 2.

### 2.3 Statistical analysis

After excluding Sample 1 participants with incomplete data for the intake SPS (47.2%) or ASI (70.7%)—a total of 72 remained. The sample was missing this data for unclear reasons; original investigators did not respond to inquiries. Remaining participants were then sub-grouped into overlapping cohorts by specific reported substances used (see Table 1) and analyzed separately. Only cohorts consisting of >75% of the total sample size were analyzed in order to maximize power. Among Sample 1 participants, >75% reported using alcohol, cannabis, and tobacco. Thus, Sample 1 participants were separated into six cohorts based on reported use of each of these three substances for both 30-day use (ASI-30) and lifetime use (ASI-LT). In addition to the five remaining Sample 1 cohorts, we examined methamphetamine ASI-30 and ASI-LT (100% of the sample) and alcohol ASI-LT for Sample 2.

**Table 1:** Demographics.

	Sample 1		Sample 2	
N	72		86	
Age (SD)	30.79 (8.49)		43.41 (10.12)	
SPS (SD)	74.14 (9.13)		67.85 (11.8)	

Race (%)				
White	72.2		42.7	
African American	17.7		32.6	
Native American	2.8		1.1	
Asian	2.8		4.5	
Hispanic	2.8		10.1	
Other/multiracial	2.8		9.0	

Education (%)				
No HS diploma	25.0		7.8	
HS diploma	27.8		22.2	
GED/trade	45.8		3.3	
Some college	1.4		41.1	
Bachelor degree	0.0		15.6	
Graduate degree	0.0		10.0	

Religion (%)				
Protestant	27.8		54.4	
Catholic	11.1		24.4	
Jewish	0.0		3.3	
Islamic	1.4		0.0	
None	30.6		11.1	
Other	29.2		6.7	

Relationship status (%)				
Single	45.7		70.0	
In relationship	12.9		21.1	
Divorced	7.0		5.6	
Separated	5.7		0.0	
Other	28.7		3.3	

Substance use detected on ASI-30/LT (% of N)				
	30-day	Lifetime	30-day	Lifetime
Alcohol	76.39	77.78	58.14	76.74
Cannabis	86.1	88.89	51.16	55.81
Tobacco	88.89	88.89	–	
Amphetamine	56.94	56.94	100	100
Cocaine	45.83	47.22	12.79	53.49
>1 Substance	66.67	93.06	77.91	90.70

94% of prison inmates using tobacco reported using 30 out of the past 30 days. Due to this extremely skewed distribution, 30-day tobacco use for Sample 1 was excluded from analysis.

Spearman's rank order correlation was used to examine the relationship between social support and both 30-day and lifetime use of alcohol and cannabis as well as lifetime tobacco use in the prison inmate sample and between social support and both 30-day and lifetime use of methamphetamine as well as 30-day use of alcohol in the MSM sample. Non-parametric analyses were used because the data did not meet the normality assumption required for parametric testing. Statistical analyses were performed using SPSS (IBM Corp, 2013).

## 3 Results

See Table 1 for baseline characteristics and demographic information. SPS internal consistency reliability for Sample 1 and Sample 2 was α = 0.88 and α = 0.93, respectively. Average SPS score and ASI-LT/ASI-30 for each cohort sub-grouped by specific substance are included in Table 2.

**Table 2:** SPS Global Score, ASI-LT/ASI-30, and correlation coefficients.

	Sample 1					Sample 2		
	ASI-LT			ASI-30		ASI-LT		ASI-30
	Alcohol	Cannabis	Tobacco	Alcohol	Cannabis	Alcohol	Methamphetamine	
n	56	64	64	55	62	66	86	
SPS (SD)	74.32(8.99)	74.56(8.69)	73.77(9.23)	74.62 (9.4)	74.56(9.07)	67.63(11.94)	67.85 (11.80)	
ASI-LT/30 (SD)	0.441(0.20)	0.384 (0.19)	0.497 (0.20)	0.484(0.37)	0.633 (0.37)	0.268(0.18)	0.398 (0.33)	0.235 (0.16)
SPS:ASI (*r*_*s*_)	−0.270⁎	−0.392⁎⁎	−0.259⁎	0.313⁎	0.070	0.175	0.063	−0.284⁎⁎

ASI-30: average # days used in past 30 days/30; ASI-LT: average # years over lifetime used ≥3 times per week/average age in years; r_s_: Spearman's rank correlation coefficient; SD: standard deviation; SPS: Social Provisions Scale.

⁎ *p* < 0.05.

⁎⁎ *p* < 0.01.

See Table 2 for Spearman's correlation values. Sample 1 demonstrated significant negative correlations between SPS and reports of lifetime use of alcohol, tobacco, and cannabis. Sample 2 demonstrated a significant negative correlation between SPS and 30-day methamphetamine use. No significant correlations were detected between SPS and 30-day cannabis use in Sample 1 or between SPS and lifetime use of methamphetamine and alcohol in Sample 2. Contrary to our expectations, we found a significant positive correlation between SPS and 30-day alcohol in Sample 1.

## 4 Discussion

The results of our analyses of two separate samples entering substance use treatment are mixed in relation to the hypothesis that perceived social support is inversely related to substance use frequency. In line with our hypothesis, perceived social support for prison inmates was inversely correlated with lifetime alcohol, cannabis, and tobacco use, and perceived social support for MSM was inversely correlated with 30-day methamphetamine use. However, there was no significant correlation between social support and 30-day cannabis use in Sample 1 or between social support and lifetime methamphetamine and alcohol use in Sample 2. Unexpectedly, perceived social support was positively correlated with 30-day alcohol use in Sample 1. Based on these exploratory correlational results, our hypothesis that perceived social support is inversely correlated with substance use in socially marginalized populations is partially supported, but results varied by population and by type and timeframe of substance used.

Our findings contribute to limited literature inversely linking social support to substance use in marginalized populations (Averna & Hesselbrock, 2001; Brick et al., 2018; Buttram, Kurtz, & Surratt, 2013), including mixed results (Spohr, Suzuki, Marshall, Taxman, & Walters, 2016). Although 30-day alcohol use and SPS in Sample 1 were significantly correlated in the opposite direction from what was hypothesized, this is not necessarily at odds with previous findings. Zywiak et al. (2009) found that while general support and social network size were inversely related to pretreatment days using drugs, social network substance involvement *positively* correlated with pretreatment *drinking* days. Because alcohol is less stigmatized than other drug use due to its legal status in the United States, the significant positive correlation in our sample might be driven by the influence (Valente, Gallaher, & Mouttapa, 2004) and selection (Bullers, Cooper, & Russell, 2001) of alcohol-using social networks—thus reflecting acceptable “social drinking”. Of note, the SPS does not distinguish the source of perceived support (e.g., family, treatment providers, or drinking versus non-drinking peers), while source of support has been shown to be a significant factor in addiction research (Litt, Kadden, Kabela-Cormier, & Petry, 2009; Best et al., 2016; Frings & Albery, 2015; Bathish et al., 2017). Finally, a clinical diagnosis of alcohol use disorder is more likely in those with higher lifetime use of alcohol (Dawson, Goldstein, Chou, Ruan, & Grant, 2008), and higher lifetime use *was* significantly correlated with lower perceptions of social support in Sample 1. As the ASI is not a diagnostic assessment, we were unable to determine who in Sample 1 was entering treatment for alcohol use disorder, specifically, versus another substance use disorder in the setting of subsyndromal alcohol use.

Our analysis has several additional limitations. Due to missing data and limited sample sizes, we were technically underpowered (Hulley, Cummings, Browner, Grady, & Newman, 2013); larger studies are required to confirm our results. Because results are correlational, no causal relation can be concluded. While we aimed to investigate marginalized populations, the samples we investigated consisted of all male participants—which limits generalizability. Recall bias may have impacted our null results for 30-day use in Sample 1, as researchers measured “typical 30-day use” prior to incarceration rather than use over the actual 30 days prior to assessment. Lastly, and our analysis did not take into account any confounding factors that may explain variation in results. For example, sociodemographic variables, comorbid psychiatric diagnoses, time incarcerated, treatment status, or other contextual factors may have impacted perceptions of social support (e.g., the legal status of cannabis in the United States is very different now than when Sample 1 data were collected).

Our correlational results are inadequate in characterizing the complex relationship between social connections and addictive substances in socially stigmatized populations. Further investigation is warranted, including prospective experimental designs aimed at identifying modifiable aspects of social support, substance use, and other treatment targets. Our findings, which suggest a quantifiable inverse relationship between perceived social support and frequency of substance use, support a growing body of literature aimed at moving away from intrapersonal, acute care models and toward treatment models that integrate families, social networks, and communities into the recovery process (Alexander, 2012; White, 2009).

## Funding

Department of Veterans Affairs Clinical Science Research & Development. Federal Award Identification Number IK2CX001495. Funding source had no involvement in study design, collection, analysis, or interpretation of data, writing the manuscript, or the decision to submit the manuscript for publication.

## Declaration of Competing Interest

The authors do not have any conflicts of interest.
